# *Araneibatrus gracilipes* gen. et sp. n., a remarkable Batrisitae (Coleoptera, Staphylinidae, Pselaphinae) from P. R. China

**DOI:** 10.3897/zookeys.69.740

**Published:** 2010-11-18

**Authors:** Zi-Wei Yin, Li-Zhen Li, Mei-Jun Zhao

**Affiliations:** Department of Biology, Shanghai Normal University, 100 Guilin Road, Shanghai, 200234, P. R. China

**Keywords:** Coleoptera, Staphylinidae, Pselaphinae, *Araneibatrus gracilipes*, new genus, new species, key, China, taxonomy

## Abstract

Araneibatrus gracilipes **gen. n.** and **sp. n.** from South China is described and illustrated. The systematic position of the genus is discussed.

## Introduction

A remarkable species with extremely elongate antenna and legs was discovered among the pselaphines collected from Nan’ling National Nature Reserve, Guangdong Province, South China, by use of a Tullgren Funnel. Based on a combination of following characters, this species was recognized as new and representing a unit for which a new genus belonging to Tribasodes genus-group of subtribe Batrisina Reitter, 1882 is erected: 1) extremely elongate antenna with three-segmentedclub ; 2) pronotum with a pair of minute lateral denticles, and with discal and antebasal spines; 3) each elytron with three basal foveae; 4) tergite IV with paratergites reduced to a pair of lateral triangular plates.

## Material and methods

The material examined was extracted from soil samples by use of a Tullgren Funnel. Dissections were made in 75% ethanol, dissected parts were mounted in Euparal on plastic slides. Photo of habitus taken with a Canon EOS 40D Camera mounted with MP-E 65 mm Macro Photo Lens. Line drawings made with Adobe Illustrator CS2.

Type material deposited in the Insect Collections of Shanghai Normal University, China (SNUC)

The terminology of foveal system mainly follows Chandler (2001).

## Taxonomy

### Araneibatrus

Yin & Li gen. n.

urn:lsid:zoobank.org:act:325B9203-6A45-4A82-AFBC-CFCBCC73CE3D

#### Type species.

Araneibatrus gracilipes Yin et Li, sp. n.

#### Diagnosis.

Head slightly elongate, sides round. Eyes situated at anterior half of head. Antenna conspicuously elongate, club loosely three-segmented, terminal segment large. Pronotum with pairs of tubercles, a pair of lateral antebasal foveae, a pair of outer basolateral foveae and a pair of inner basolateral foveae. Each elytron with three basal foveae, discal stria present. Subhumeral elytral fovea present, indistinct. Legs conspicuously elongate. Abdominal segments successively narrower posteriad, with round apex. Segment IV large. Tergite IV with paratergites forming a pair of triangular plates. Aedeagus elongate, basal bulb large, with flattened membrane and hook-like sclerite, median lobe elongate and well sclerotized, with elongate dorsal apophysis.

#### Description.

 Head with vertexal sulcus absent. Vertexal foveae small. Eyes relatively small, convex. Antennae 11-segmented, conspicuously elongate, with thick scape and loosely three-segmented club, terminal antennomere large. Pronotum nearly hexagonal, with pair of lateral antebasal foveae, pair of outer basolateral foveae, and pair of inner basolateral foveae. Elytron with three basal foveae, subhumeral elytral fovea present. Sutural stria absent, discal stria faint, marginal stria present. Venter with pair of lateral mesoventral foveae, pair of lateral mesocoxal foveae, and pair of lateral metaventral foveae. Abdomen moderately narrow and round apically. Tergite IV large. Paratergites IV present as pair of triangular plates.

#### Remarks.

Members of Araneibatrus can be distinguished from other Asian genera of Tribasodes genus-group in their conspicuously elongate antennae and legs; metatrochanter without protuberance and the structure of aedeagus. Though the new genus is tentatively placed in Tribasodes group, we recognize that the metacrochanter without protuberance and form of aedeagus is like in the other members of Batrisocenus group.

#### Bionomics.

 Only one male specimen was collected during a long-term investigation on the soil beetles of Nan’ling National Nature Reserve, indicating that this is a rare species or that we have not found a method of finding individuals.

#### Distribution.

Guangdong Province (South China)

#### Etymology.

 The generic name is derived from the Latin word “*araneosus*”, meaning “spider-like”, and combined with an arbitrary rearrangement of Batrisus Aubé, 1833, the type genus of Batrisitae. Gender masculine.

#### Araneibatrus gracilipes sp. n.

urn:lsid:zoobank.org:act:2C745861-8645-468E-BA48-EAC2B6D88643

**Figure F2:**
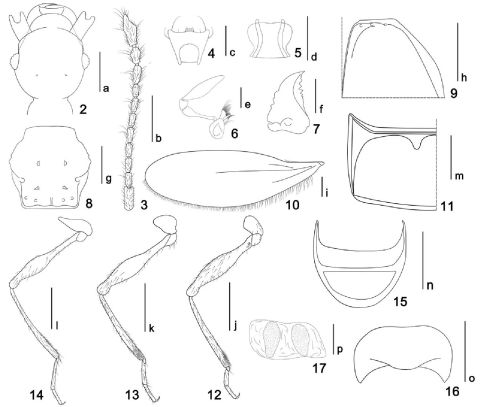
**Figures 2–17.** Araneibatrus gracilipes gen. et sp. n. **2** Head, in dorsal view **3** Right antenna **4** Labrum **5** Labium **6** Left maxilla **7** Left mandible **8** Pronotum **9** Anterior half of right elytron **10** Left hind wing **11** Left half of tergite IV **12** Foreleg **13** Midleg **14** Hindleg **15** Tergite VIII **16** Sternite VIII **17** Sternite IX. Scales: c, d, e, f, p = 0.1 mm; a, g, h, m, n, o = 0.2 mm; i = 0.3 mm; b, k, l, m = 0.4 mm.

Figures 1 [Fig F2] 20 

##### Type material.

HOLOTYPE male, China: Guangdong Province, Nan’ling N. R., No. 6 Forest-Road,24°56'34"N; 113°01'26"E, alt. 1,388 m, Oct. 2009, Lei GAO leg.

##### Description.

 Body (Fig. 1) length 2.73 mm, width 1.22 mm. Reddish–brown, appendages slightly lighter in color.

**Figure:**
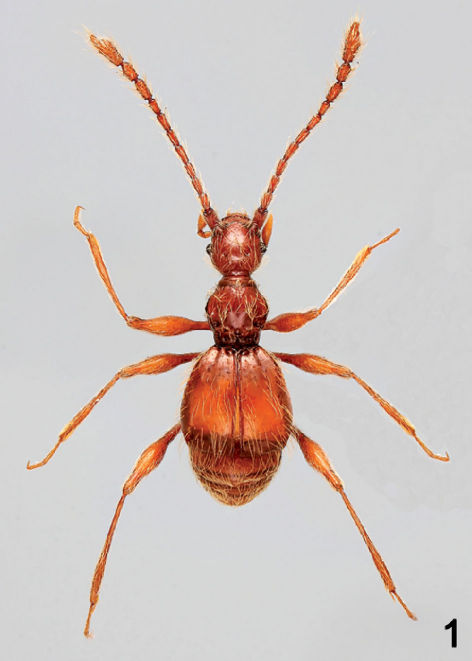
**Figure 1.** Habitus of Araneibatrus gracilipes gen. et sp. n.

Head (Fig. 2) slightly elongate, sides round, smooth, covered with short setae; clypeus short, with rounded anterior margin, densely covered with short setae; frons narrower, moderately depressed in median part, convex at antennal tubercles, sparsely covered with short setae; center of vertex shlightly raised, sparsely covered with short setae, with pair of small vertexal foveae; postgena rounded laterally, covered with long setae on both sides; gular area smooth, gular foveae merged into a single pit, gular carina faint. Eyes relatively small, semispherical, each composed of about 35–40 facets; mouthparts normal in structure; labrum (Fig. 4) nearly trapezoidal, narrower posteriad, anterior margin with four specialized setae; mandible (Fig. 7) large, outer margin arcuate, with several blunt denticles near its middle, cutting edge with three to four big teeth and about ten smaller teeth; labium (Fig. 5) slightly wider than long, with anterior margin concavely emarginate, sides round, constrict at basal 1/3. Maxillary palpi (Fig. 6) large and elongate, with palpomere I tiny, indistinct, II pedunculate, thickened distally, III short, nearly triangular, IV largest, covered with short setae, about as long as combined length of palpomeres I to III, nearly fusiform, about three times as long as wide, widest near its basal 2/5. Antenna (Fig. 3) conspicuously elongate and slender, antennomeres elongate; scape cylindrical, large and thickened, about 2.5 times as long as wide, with swollen apex; pedicel about twice as long as wide, gradually thickened anteriad; III to V successively narrower and more elongate; VI narrower than V, short, 3/4 times as long as V; VII slightly longer than VI, but narrower; VIII the smallest, about 3/4 times as long as VII; IX to XI loosely clubbed, successively wider; IX about 1.25 times as long as VIII, slightly asymmetrical, widest near its middle; X slightly shorter but wider than IX, thickened, inner margin with distinct process; XI largest, about 1.8 times as long as X, narrowest at base, gradually widened toward its middle, then narrowed toward apex, lateral margin irregular with several small processes. Basolateral margins of antennomere II, VIII, IX, X and XI protuberant, forming tiny spine-like process (this character may only occur in males).

Pronotum (Fig. 8) nearly hexagonal, slightly wider than head and about as long as wide, widest at middle; sides strongly sclerotized, shallowly and roughly dentate, disc with two pairs of tubercles. Elytrae (Fig. 9) convex dorsally, both together slightly wider than long, sparsely covered with hairs. Hind wing (Fig. 10) large and elongate, widest at middle, with round apex. Venter strongly convex in apicomedian part, widest at apex. Legs normal in structure, conspicuously elongate and slender; foreleg (Fig. 12) with femor widest near middle, with tuft of short setae near base, tibia densely covered with short setae at base; midlegs (Fig. 13) similar to forelegs, but slenderer; hindlegs (Fig. 14) even more slender than midlegs, with femora widest near basal 1/3.

Abdomen with tergite IV (Fig. 11) large, with deep basal excavation, discal carinae absent. Paratergites IV reduced to a pair of triangular plates; following segments successively shorter and narrower, setose; tergite VIII (Fig. 15) concavely excavate on posterior margin, with round apex; sternite VIII (Fig. 16) transverse, with strongly emarginate anterior margin, posterior margin slightly concave. Sternite IX (Fig. 17) semi-membranous, transverse, with two weakly sclerotized plates.

Aedeagus (Figs. 18–20) well-sclerotized, ventral stalk elongate and slender, broadened basally, slightly curled to the right; dorsal apophysis elongate and slender, connected with ventral stalk at base, strongly curled to the left in ventral view; hook-like sclerite erect just behind ventral stalk and curled to the left in ventral view; the strongly expanded semi-sclerotized membrane derived from the end of basal bulb widest near its middle and then gradually narrowed and ending to left in ventral view; basal bulb large, with round base.

**Figure:**
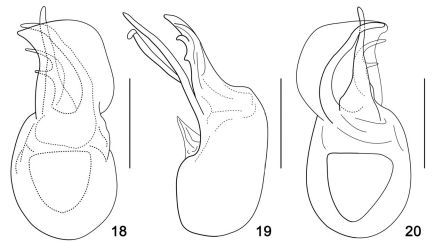
**Figures 18–20.** Aedeagus of Araneibatrus gracilipes gen. et sp. n. **18** Aedeagus in dorsal view **19** Same, lateral view **20** Same, ventral view. Scales = 0.2 mm.

##### Remarks.

The new species is distinct by its conspicuously elongate and slender antennae and legs. A number of external characters of the new genus, such like the roughly dentate sides of the pronotum, each elytron with three basal foveae, tergite IV with a pair of triangular paratergites, provide evidence for placement of Araneibatrus gracilipes gen. et sp. n. in the Tribasodes genus-group. The presence of the movable dorsal apophysis of the aedeagus suggests a relationship to the Batrisocenus-group. Thus, according to S. Nomura (pers. comm.), the new taxon appears intermediate between these two closely related groups.

##### Etymology.

The name is derived from the Latin words, “*gracilis*”, meaning “slender” and “*lipes*”, meaning “leg, limb”.

## Supplementary Material

XML Treatment for Araneibatrus
